# 1698. Activity of ceftolozane/tazobactam *in vitro* against Gram-negative isolates from pediatric patients: SMART 2017-2021

**DOI:** 10.1093/ofid/ofad500.1531

**Published:** 2023-11-27

**Authors:** Xinli Mu, Ying Fu, Pengcheng Li, Yunsong Yu

**Affiliations:** Sir Run Run Shaw Hospital, School of Medicine, Zhejiang University, Hangzhou, Zhejiang, China; Sir Run Run Shaw Hospital, School of Medicine, Zhejiang University, Hangzhou, Zhejiang, China; MRL GMA, MSD China, Shanghai, Shanghai, China; Sir Run Run Shaw Hospital, School of Medicine, Zhejiang University, Hangzhou, Zhejiang, China

## Abstract

**Background:**

The study aims to evaluate the *in vitro* activity of ceftolozane/tazobactam (C/T) and other common-used antibiotics against clinical Gram-negative bacterial (GNB) isolates obtained from Chinese pediatric patients using data from The Study for Monitoring Antimicrobial Resistance Trends (SMART).

**Methods:**

From 2017-2021, 554 Gram-negative isolates were collected from 16 pediatric departments across China. GNB isolates from pediatric departments were mainly derived from bloodstream (n = 135), intraperitoneal (n = 129), lower respiratory tract (n = 198) and urinary tract (n = 92) infections (**Figure 1**). The minimum inhibitory concentrations (MICs) were tested using a Trek Diagnostic System (Thermo Fisher Scientific). Susceptibility was determined by CLSI broth microdilution and interpreted with CLSI M100 (2021) breakpoints.

**Figure:**
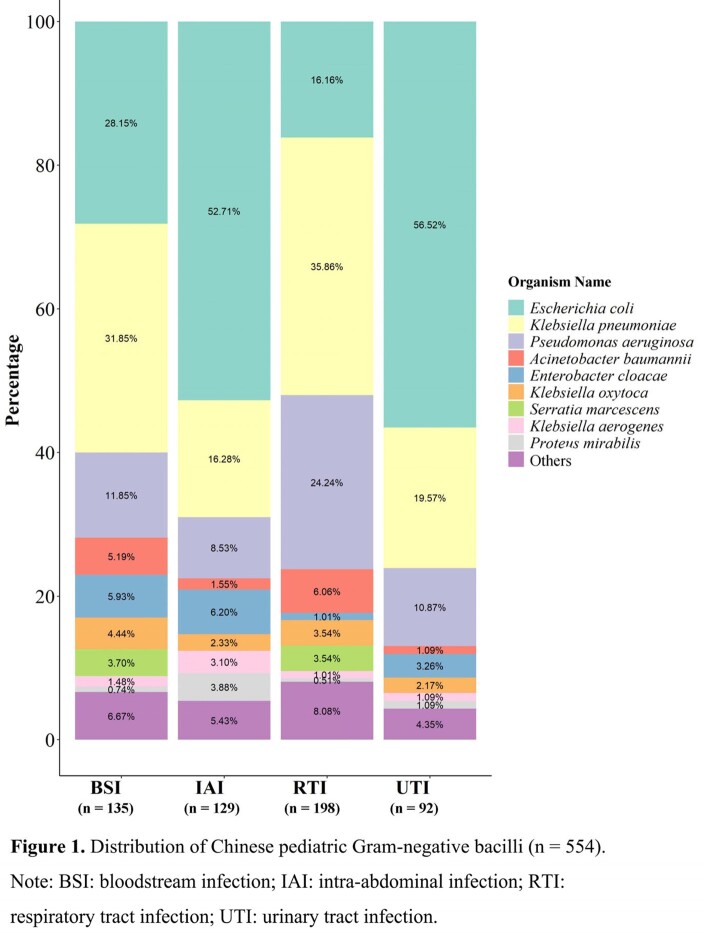

**Results:**

*E. coli* (34.30%) and *K. pneumoniae* (27.62%) were the most common pathogens, followed by *P. aeruginosa* (15.34%). Susceptibility of species with more than 20 isolates were showed in **Table 1**. The susceptibility of *P. aeruginosa* to C/T was 89.41%, which was the highest among beta-lactams and was second only to amikacin. The susceptibilities of *E. coli* and *K. pneumoniae* isolates to C/T were 92.63% and 58.17%, respectively. When exclude carbapenem-resistant *E. coli* and *K. pneumoniae*, the susceptibility to C/T increased to 96.17% and 86.14%. C/T showed similar activities to *E. coli* and *K. pneumoniae* isolated from pediatric patients < 1 year (**Table 2**). Thirteen *P. aeruginosa* isolates were collected from patients < 1 year and 13/13 were susceptible to C/T (**Table 3**).

**Figure:**
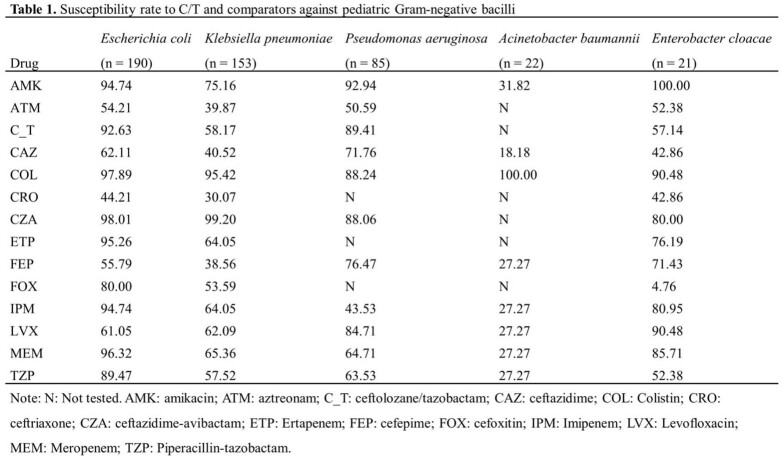

**Figure:**
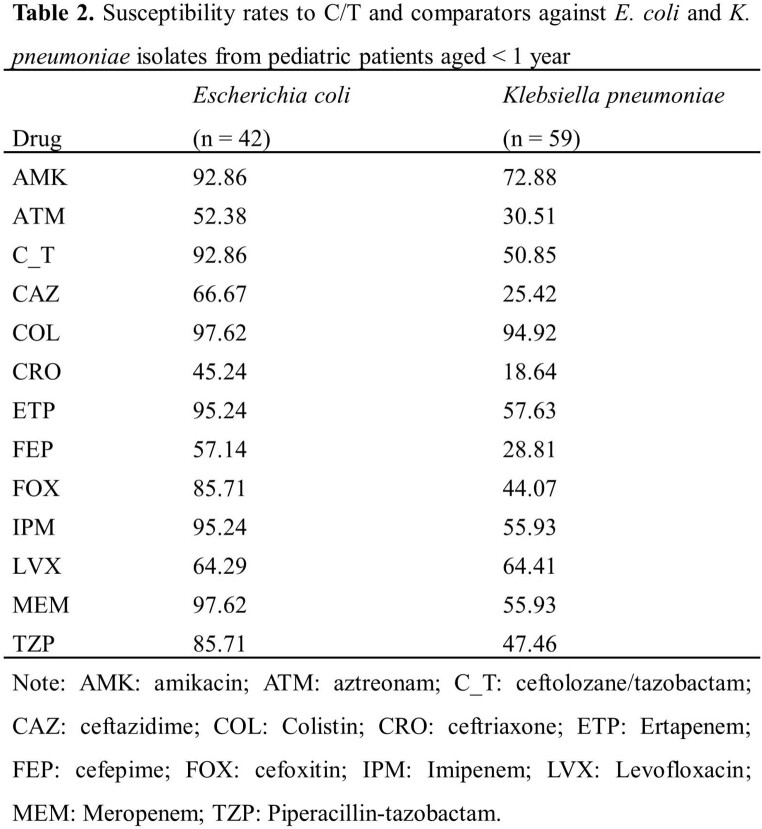

**Figure:**
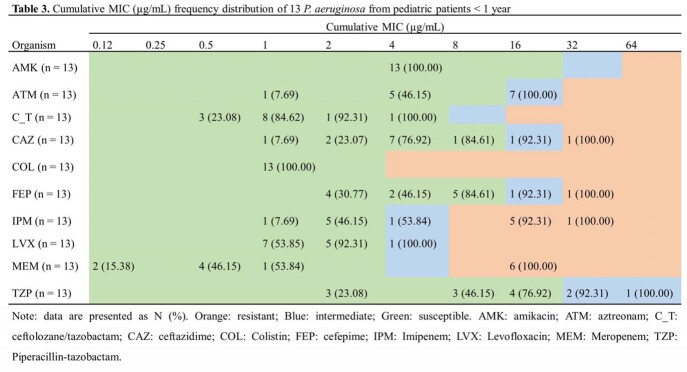

**Conclusion:**

*P. aeruginosa* and *E. coli* obtained from pediatric patients in China showed a high susceptibility to C/T. When excluding carbapenem resistant isolates, C/T also showed good activities against *K. pneumoniae*.

**Disclosures:**

**Pengcheng Li, M.D.**, MSD China: Employee of MSD China

